# Pneumoperitoneum and peritonitis secondary to perforation of an infected bladder

**DOI:** 10.1016/j.ijscr.2021.105783

**Published:** 2021-03-16

**Authors:** Eric Bergeron, Daniel Lewinshtein, Lionel Bure, Chantal Vallee

**Affiliations:** a Departments of Surgery, Charles LeMoyne Hospital, Greenfield Park, Canada; b Medical Imaging, Charles LeMoyne Hospital, Greenfield Park, Canada; c Internal Medecine, Charles LeMoyne Hospital, Greenfield Park, Canada

**Keywords:** Peritonitis, Pneumoperitoneum, Cystitis, Bladder perforation, Indwelling catheter, Case report

## Abstract

**Introduction and importance:**

Spontaneous urinary bladder rupture is a rare complication of urosepsis. Its co-occurrence with pneumoperitoneum is even more unusual.

**Case presentation:**

A 73-year-old patient presented with acute retention with mild lower abdominal pain and difficulty with urinary voiding and cystitis. He was treated with bladder catheter and antibiotics. After one month, he suddenly developed peritonitis and shock. Pneumoperitoneum was observed on a chest x-ray. An emergent laparotomy was performed and a perforation of the bladder secondary to necrosis of part of the wall was found and resected. The patient recovered satisfactorily after the surgical intervention.

**Clinical discussion:**

Spontaneous bladder rupture is a life-threatening condition that could be missed. Surgical intervention is mandatory to rule out other more probable causes of peritonitis and to manage the bladder perforation itself.

**Conclusion:**

Pneumoperitoneum is rarely secondary to a bladder perforation. Immediate surgical intervention is required in order to avoid delays in treating any intra-abdominal condition including a bladder wall perforation.

## Highlights

• Spontaneous urinary bladder rupture is a rare complication of urosepsis.
• Co-occurrence of pneumoperitoneum with a bladder perforation is very rare.
• Surgical intervention is mandatory in most cases of bladder perforations.

## 1 Introduction

Spontaneous urinary bladder rupture is a rare complication of urosepsis [1,2]. Catheterization or indwelling catheter may also be a risk or contributing factor for bladder rupture [1,[3], [4], [5], [6], [7], [8]]. Among the other associated complications with bladder perforation, the occurrence of pneumoperitoneum is very unusual [2,[4], [5], [6],^9^,^10^]. In majority of bladder perforation cases, clinical presentation, radiology imaging, and rarity lead to misdiagnosis as digestive tract perforation [2,6]. We describe a case of unsuspected bladder perforation presenting with peritonitis and free air under the diaphragm. The case is discussed, together with an updated literature review. SCARE criteria [11] have been used for this report.

## 2 Case presentation

A 73 year old male with no significant past medical history presented on September 1st 2020 at the emergency department. He had no history of bladder or prostate disease, or urinary procedure before. During the 3 weeks prior to arriving at the hospital, he complained of mild lower abdominal pain, difficulty with urinary voiding and stools every 2 or 3 days almost liquid. On examination, he was hypotensive (87/59) and his temperature was 38.4 °C. The patient looked asthenic. The abdomen was slightly distended and sensitive on the lower part. There was no defense and no rebound tenderness.

Blood levels of creatinine was 295 μmol/L (Normal: 60–105 μmol/L) and lactate was 3.8 mmol/L (Normal: 0.5–1.6 mmol/L). White blood cell count was 17,300/mm^3^ with 94% of neutrophil cells. Urinalysis was positive for the presence of bacteria, white cells and nitrites. The patient was rehydrated and started on piperacillin-tazobactam.

**Fig. 1:**
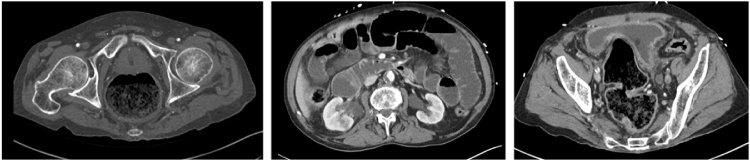
Computed tomography at admission. Left panel: Shows fecaloma and thickened rectal wall; Middle panel: Shows dilated bowel loops with no transition point; and Right panel: Shows distended bladder and edematous wall.

Computed tomography (Fig. 1) showed a rectal fecaloma, edema of the sigmoid part of the colon, slightly distended small bowel with no evidence of occlusion, and a thickened and distended urinary bladder. No diverticulum was demonstrated. Culture of urine and blood revealed the presence of *Escherichia coli*. The patient was thus treated for urosepsis and ileus.

**Fig. 2:**
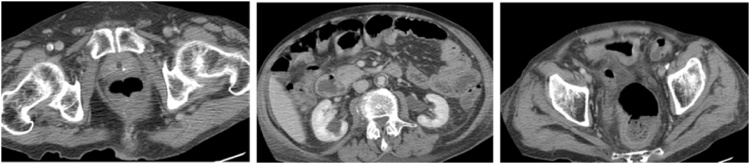
A repeated computed tomography ten days after the first CT. Left panel: Shows resolution of fecaloma; Middle panel: Shows resolution of the small bowel distension; and Right panel: Shows persistent wall thickening and air within bladder.

A bladder catheter was inserted without difficulty, and antibiotics were changed for intravenous cefazolin. A cystoscopy revealed a severe cystitis and absence of diverticulum. There was no evidence any of prostatomegaly or bladder outflow obstruction. Biospies confirmed the diagnosis of severe cystitis without any underlying pathology. The fecaloma was evacuated during the bowel preparation for colonoscopy, which was subsequently normal. A repeated computed tomography ten days later (Fig. 2) revealed the disappearance of the fecaloma, decreased distension of the small bowel, and the thickened and distended bladder that is unchanged despite the presence of a Foley catheter. There was the presence of air within the bladder.

The patient was put on parenteral nutrition owing to asthenia and inappetence. The bladder catheter was changed every week after failed attempts to void normally. As the sepsis resolved, antibiotic treatment was completed after 12 days.

**Fig. 3:**
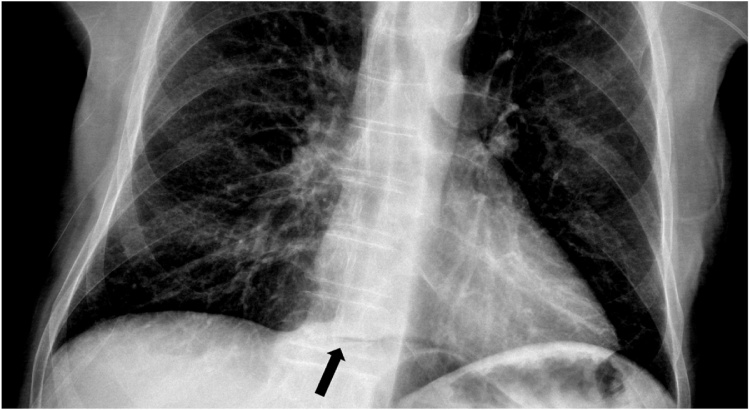
Chest X-ray showing subdiaphragmatic free air along the midline.

On October 3rd, the patient became febrile with a temperature of 39.0 °C. A chest X-ray was done to rule out a pneumonia. Pneumoperitoneum was demonstrated (Fig. 3). The patient was immediately seen by the general surgeon. The patient soon became hypotensive with systolic blood pressure less than 60. The patient remained responsive. The abdomen was not distended but showed diffuse defense, and the patient clearly had peritonitis.

**Fig. 4:**
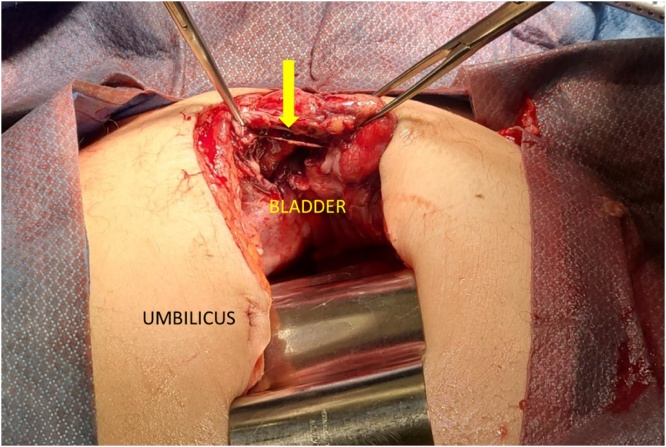
Perforation of the bladder in the Reitzius’ space.

Because of the urgency of the situation, the patient was promptly brought to the operating room with a presumed diagnosis of perforation of the small bowel. Upon opening the abdomen, a moderate quantity of free fluid with a smell of urine was noticed. There was no pus or pseudomembranes. In the Reitzius’ space, there was a 3 cm perforation of the bladder (Fig. 4). The bladder almost reached the umbilicus, despite the Foley catheter well in place. There was a marked edema of the surrounding tissues with a swollen and adherent loop of small bowel. There was no evidence of bowel obstruction or perforation. A 24-French urethral catheter was inserted, and a partial cystectomy was carried out. Culture of the abdominal fluid was done. The peritoneal cavity was cleaned thoroughly, and a peritoneal drain was left in place.

Post-operative course was uneventful. The patient was kept on piperacillin-tazobactam for 10 days. The patient rapidly tolerated food, and intestinal transit resumed within few days. He was discharged three weeks later, and the bladder catheter was changed without any problems. Three months later, the patient reported no complaints. The bladder catheter was removed, and the patients was able to void normally.

Final pathology reports revealed a severe cystitis with complete necrosis of the wall and acute perforation. Culture of the abdominal fluid grew Escherichia coli, the same germ that was found in urine upon admission.

## 3 Discussion

Spontaneous bladder rupture, defined as a perforation without external stimulation, is a very rare situation [2]. The incidence of urinary bladder rupture, from all causes, is reported to be between 0.0007% and 0.002% of hospital admissions [12]. Spontaneous bladder rupture represents only 3.4% of these cases [2]. It results mostly from an underlying pathology [1,2,10,13], and is a rare complication of urosepsis [1,2,6].

Diagnosis of bladder perforation is rarely done preoperatively [1,4,6], often delayed [7,14] or discovered only during laparotomy [1,6]. Abdominal pain or peritonitis is the usual presentation. Forty percent of bladder ruptures are intraperitoneal [3]. In the majority of cases, the presumed, but mistaken, diagnosis is digestive tract perforation [2,6,10,14], particularly in the presence of pneumoperitoneum [2,6,10]. Computed tomography usually shows free fluid [4,7,14], but pneumoperitoneum is demonstrated in 16% of the cases [9]. When diagnosis of bladder perforation is suspected, as in patients with previous bladder surgery [7], CT cystogram is the most useful diagnostic modality [2,5,7,14].

In the present case, a sudden onset of peritonitis and shock prompted rapid intervention without further investigation. Perforation of intestine was the presumed diagnosis, and the presence of pneumoperitoneum supported this assumption. The absence of pseudomembranes onto the peritoneal surfaces demonstrated that perforation occurred very recently. The presence of free air may be the consequence of urethral catheterization or urinary infection, secondary to gas forming bacteria [11], and both these situations are likely in this case. Only few milliliters of free air may be demonstrated even on standard chest X-ray [15].

The perforation of the bladder was secondary to the bladder necrosis. The catheter does not appear to be the cause of the necrosis of the bladder wall, even if it were exchanged the day before. Rupture may even occur in the absence of manipulation in the case of urosepsis [1,2,6]. The presence of an indwelling catheter or catheterization itself certainly is a contributing factor [1,[3], [4], [5], [6], [7], [8]] or a precipitating factor [10] in this case. However, the size of the perforation (Fig. 4) was too large to be due only to the passage of the catheter. Also, the catheter was well positioned into the bladder at the operation and could not have caused the perforation.

In occasional cases of stable patients with a confirmed diagnosis of bladder rupture [2,4,5,7,10,14], conservative treatment with bladder drainage, antibiotics and supportive measures may be successfully attempted [4,7]. Even with proper diagnosis, surgical intervention is mandatory if the patient is unstable, deteriorating or clearly in peritonitis [2,5]. As in our case, if there is bladder necrosis [5,7,10,13], it necessitates the removal of the necrotic part of the bladder, in addition to just closing the perforation and washing the peritoneal cavity.

Mortality associated with spontaneous bladder rupture is reported to be as high as 50% [14]. With the increase in the elderly and high-risk population with indwelling bladder catheter, which is associated with urinary tract infection and bladder mucosal damage [6], incidence of bladder perforation is expected to rise. However, rate of catheter-related bladder ruptures, although unknown, will probably remain stable and low [5].

Although differential diagnosis must certainly include spontaneous bladder rupture in patients with cystitis with or without indwelling catheter [4,8], investigation should not cause further delays and preclude prompt surgical intervention in case of presumed digestive tract perforation or peritonitis [2,5,6,10,13,16]. Except for occasional cases of intraperitoneal bladder perforation that are stable and manageable without surgery [4,7], peritonitis from other causes than bladder perforation has to be ruled out [[1], [2], [3],^6^,^10^,^13^,^16^]. Additionally, surgical intervention remains the mainstay of treatment for bladder perforation in the majority of cases [7].

## 4 Conclusions

Spontaneous bladder rupture may occasionally occur in patients with acute cystitis with or without bladder catheterization or indwelling catheter. Patients may present with peritonitis and even with pneumoperitoneum. Prompt surgical intervention is mandatory in most cases to rule out other and more probable causes of peritonitis, and to repair the bladder perforation itself. Delays in treating this life-threatening condition should be avoided.

## Declaration of Competing Interest

No conflict of interest to declare.

## Sources of funding

No funding was necessary.

## Ethical approval

Not necessary for a case report by our institution.

## Consent

Consent was obtained by the patient. There is no possibility to identify the patient from the text and images.

## Author contribution

EB, DL, CV managed the case. EB reviewed the literature. EB, DL wrote the paper. LB provided, and interpreted radiology material. EB, DL, LB, CV reviewed the manuscript.

## Registration of research studies

Not applicable.

## Guarantor

EB accept responsibility for this publication.

## Provenance and peer review

Not commissioned, externally peer-reviewed.
